# On the Present State of Medicine in the United States

**Published:** 1836-10

**Authors:** Robley Dunglison

**Affiliations:** Professor of the Institutes of Medicine and Medical Jurisprudence in Jefferson College, Philadelphia


ON THE

### PRESENT STATE OF MEDICINE IN THE UNITED STATES;

By Robley Dunglison, m.d., Professor of the Institutes of Medicine and Medical Jurisprudence in Jefferson College, Philadelphia.

### ART. I. OF THE MEDICAL INSTITUTIONS.

### A. MEDICAL COLLEGES.

When the continent of America was first visited by emigrants from Great Britain, a few practitioners of medicine doubtless formed part of the body; in the first instance, perhaps, leaving the mother country with the same prospects as their fellow-emigrants, but devoting themselves likewise to the duties of their profession, as occasion required. Of the condition of medicine at this early period of the colonial history, we know nothing. It would seem, however, that in New England, for many years after the first settlement of the country, it was deemed indispensable for clergymen to acquire a knowledge of practical medicine; and we find that, not only did they prescribe for the afflicted, but they entered into medical controversies, and wrote treatises on the diseases of the country.

It was not until a short time before the revolution that any attempt was made to establish a medical school in the colonies. As early as the year 1638, the College of Harvard was founded at Cambridge, in New England; and, in 1691 and 1700, William and Mary College in Virginia, and Yale College in Connecticut, were respectively established. Many of the alumni of these institutions, and of Princeton, New Jersey, founded in 1746, visited Europe to attend the medical lectures, in Edinburgh more especially, and, after having graduated, returned to America to practise their profession. Nearly all the most eminent physicians and surgeons who commenced practice before the revolution received their medical education in Europe, and a large portion of them emigrated from Great Britain; for it would appear that it was not until the political bonds between the two countries had been severed, that the conviction was entertained that the science of medicine could be adequately taught in America.

In the year 1750, the body of Hermanus Carroll, a criminal, who had been executed for murder, was dissected in the city of New York, by Dr. John Bard and Dr. Peter Middleton, two of the most eminent physicians of the day; and this would seem to have been the first effort made in the United States for the purpose of imparting medical knowledge by the dissection of the human body of which there is any record. Some years after this, a course of lectures on anatomy and surgery, accompanied by dissections of the human body, was delivered at Newport, Rhode Island, by Dr. William Hunter, a native of Scotland, and a near relation of William and John Hunter. He was educated at Edinburgh, under the first Monro; went to Rhode Island about the year 1752, and gave lectures on anatomy, on the history of anatomy, and on comparative anatomy, in the years 1754, 5, and 6, to which not only the medical students and physicians, but all the literary gentlemen of the town, were invited.

### I. UNIVERSITY OF PENNSYLVANIA. (PHILADELPHIA.)

The first conception of a plan for establishing a medical school in America appears to have been formed by Dr. William Shippen and Dr. John Morgan, both native Americans, while engaged in their studies in Europe. In the year 1762, the former of these gentlemen, in the introductory lecture to a private course of anatomy, announced his belief in the expediency and practicability of founding a medical school in Philadelphia. In 1765, Dr. Morgan, on his return from Europe, laid before the trustees of the College of Philadelphia, which had then been in existence as a collegiate establishment about ten years, a plan for the institution of medical professorships, in connexion with the institution under their direction. The plan, strongly recommended by several influential friends of the College in England, was adopted by the trustees, who appointed Dr. Morgan to the chair of the Theory and Practice of Physic. In the same year, Dr. Shippen was chosen professor of Anatomy and Surgery; and, for a short period, lectures were delivered by these two professors on the various branches of the science then deemed essential in a course of medical instruction. In 1767, a system of rules was adopted for the organization of the new school. In 1768, Dr. Adam Kuhn was appointed professor of Materia Medica and Botany, and Dr. Thomas Bond of Clinical Medicine; and, on the 21st of June, 1768, a medical "commencement" was held, at which the degree of bachelor of medicine was conferred upon ten individuals. In 1769, the chair of Chemistry was added, to which the distinguished Benjamin Rush was appointed.

As the school advanced, additional professorships were created; but it had not been long in action before a rival institution was established and connected with the university; a circumstance which gave rise to much contention, but was finally allayed in 1791, by a union of the two schools.

The following is the present organization of the medical department of this university.

The faculty consists of seven professors, independently of Dr. Physick, who holds the station of Emeritus Professor of Surgery and Anatomy, but does not officiate.

1. Nathaniel Chapman, M.D., Prof. of the Theory and Practice of Medicine.
2. Robert Hare, M.D., Professor of Chemistry.
3. William Gibson, M.D., Professor of Chemistry.
4. William E. Horner, M.D., Professor of Anatomy.
5. Samuel Jackson, M.D., Professor of the Institutes of Medicine.
6. George B. Wood, M.D., Professor of Materia Medica and Pharmacy.
7. Hugh L. Hodge, M.D., Professor of Midwifery and the Diseases of Women and Children.

The following table exhibits the number of students who have attended the lectures in this institution, and the number of graduates in each year, from the winter of 1810-11 to that of 1835-6, inclusive.

| Winter. | Matriculates. | Graduates. | Winter. | Matriculates. | Graduates. |
|---|---|---|---|---|---|
| 1810-11 | 406 | 65 | 1823-24 | 424 | 96 |
| 1811-12 | 387 | 70 | 1824-25 | 487 | 111 |
| 1812-13 | 349 | 61 | 1825-26 | 440 | 114 |
| 1813-14 | 345 | 62 | 1826-27 | 441 | 131 |
| 1814-15 | 319 | 44 | 1827-28 | 409 | 133 |
| 1815-16 | 388 | 70 | 1828-29 | 362 | 109 |
| 1816-17 | 436 | 74 | 1829-30 | 421 | 127 |
| 1817-18 | 465 | 87 | 1830-31 | 410 | 151 |
| 1818-19 | 422 | 102 | 1831-32 | 386 | 134 |
| 1819-20 | 330 | 78 | 1832-33 | 367 | 117 |
| 1820-21 | 325 | 66 | 1833-34 | 432 | 145 |
| 1821-22 | 357 | 77 | 1834-35 | 390 | 135 |
| 1822-23 | 455 | 101 | 1835-36 | 398 | 132 |

In the twenty-six years, the number of students has consequently amounted to 10,331, and of graduates to 2592; the average number per annum of the former being 398, and of the latter 100. The great increase of the graduates over the matriculates since the year 1810 is ascribed by the faculty, in a printed address by Professor Wood, recently published by their direction, to two chief causes: first, the establishment of other schools, the pupils of which are permitted to become candidates for a degree in the university of Pennsylvania, after attending one full course of lectures, instead of two courses in the latter; and, secondly, the greater diffusion of knowledge through the community, which renders a degree desirable as an evidence of qualification to practise, where formerly it was deemed of little consequence.

II. COLLEGE OF PHYSICIANS AND SURGEONS, NEW YORK. (NEW YORK.)

This was the second medical school instituted in America, in the year 1768. Drs. Clossy, Bard, Jones, Middleton, Smith, and John V. B. Tennent were the first professors. The school was connected with King's, now Columbia College, and, in 1769, the degree of bachelor of medicine was conferred upon Samuel Kissam and Robert Tucker, the first graduates. The school had been in existence but a few years, when its labours were interrupted by the revolutionary war. On the return of peace various attempts were made to revive it, but, owing to feuds and collisions among the members of the profession, every effort was vain. Private lectures were, however, delivered by many respectable teachers; until, in 1792, a new organization of a medical school was effected by the trustees of Columbia College, but the advantages accruing from it were deemed by no means commensurate with their expectations; and, accordingly, it was deemed expedient to grant a charter, establishing the College of Physicians and Surgeons, in March, 1807. A spirit of rivalry now commenced between the schools, which led to the most unfortunate results; so that, in 1811, the regents were induced to remodel the College of Physicians and Surgeons, with a view to their union with the Medical Faculty of Columbia College, which was effected in 1813. For about seven years from this period the College of Physicians and Surgeons went on prosperously; but difficulties arose; charges of serious import were brought against the professors, men undoubtedly of talent and respectability: these charges (to use the language of a medical historian of the times,) on investigation by the regents, in March, 1825, were declared to be unsubstantiated, and were pronounced by that body, in an elaborate report, to have arisen from jealousy and professional rivalry. "Broils and contention, nevertheless, continued, and the opposition persisted systematically in their purpose. In April, 1826, the professors, wearied with unavailing attempts to silence the opposition, came to the conclusion that 'it would best consist with their own self-respect' to withdraw altogether from the institution; and accordingly they tendered the resignation of their professorships and offices. The Board of Regents accepted their resignations, April 17, 1826, and presented their thanks 'for the faithful and able manner in which they had filled their respective chairs as instructors and lecturers in the said college.'"

The professors at that time were Drs. Hosack, Macneven, Samuel L. Mitchell, Mott, and Francis; Dr. Post having previously given in his resignation. Through the agency of these gentlemen, an offset from Rutger's College, at New Brunswick, in New Jersey, was established in New York; but, although the number of students frequenting its halls was considerable, it was necessarily abandoned, owing to the legislature of New York refusing it a charter.

The present faculty are as follows:

1. John Augustine Smith, M.D., Professor of Physiology.
2. Alexander H. Stevens, M.D., Professor of the Principles and Practice of Surgery.
3. Joseph M. Smith, M.D., Professor of the Theory and Practice of Physic and Clinical Medicine.
4. Edward Delafield, M.D., Professor of Obstetrics and Diseases of Women and Children.
5. John B. Beck, M.D., Prof. of Materia Medica and Medical Jurisprudence.
6. John Torrey, M.D., Professor of Chemistry and Botany.
7. Valentine Mott, M.D., Professor of Operative Surgery, and Surgical and Pathological Anatomy.
8. John R. Rhinelander, M.D., Professor of Anatomy.

James Quackenbush and James Bolton, Demonstrators.

The number of students, in the session 1835-6, was 124; the number of graduates, session 1834-5, was nineteen.

### III. MEDICAL SCHOOL OF HARVARD. (BOSTON.)

This school was first suggested by private munificence. Dr. Ezekiel Hersey, of Hingham, in Massachusetts, who died in 1770, bequeathed one thousand pounds, and his widow, at her decease, a like sum, to be applied to the establishment of a professorship of anatomy and surgery. His brother, Dr. Abner Hersey, of Barnstaple, who died in 1786, and Dr. John Cuming, of Concord, also gave five hundred pounds each, for the same object; and William Erving, Esq., of Boston, bequeathed one thousand pounds towards the endowment of an additional professorship. In 1780, Dr. John Warren, the father of the present professor of anatomy, whilst surgeon of a military hospital in Boston, commenced a course of anatomical lectures, which were attended, in the following year, by the students of the university. Dr. Warren furnished a plan for a medical school, which was adopted, in 1782, by the corporation of Harvard College. He was appointed professor of anatomy and surgery; Dr. Benjamin Waterhouse, professor of the theory and practice of physic; and Dr. Aaron Dexter, professor of chemistry. In consequence of the greater advantages likely to accrue from the lectures being delivered in the city of Boston, the corporation and board of overseers of Harvard University deemed it expedient to remove the medical school to that city, which was done in 1810.

The present faculty are:

1. John C. Warren, M.D., Professor of Anatomy and the Operations of Surgery.
2. John W. Webster, M.D., Professor of Chemistry.
3. Walter Channing, M.D., Professor of Midwifery and Medical Jurisprudence.
4. Jacob Bigelow, M.D., Professor of Materia Medica.
5. George Hayward, M.D., Professor of the Principles of Surgery and Clinical Surgery.

9. James Jackson, M.D.,* and John Ware, M.D., Professors of the Theory and Practice of Physic and Clinical Medicine.

The number of students, session 1835-6, was 118.

### IV. DARTMOUTH COLLEGE, NEW HAMPSHIRE. (HANOVER.)

This was the fourth medical school instituted in the United States. It is situated at Hanover, New Hampshire, and was founded by Dr. Nathan Smith, the father of the present professor of surgery in the university of Maryland. In 1798, Dr. Smith was appointed sole professor of the school, and for twelve years he gave lectures on the various departments of medicine, excepting two courses, in which he was assisted in the department of chemistry.

The present faculty are three in number:

1. Reuben Dimond Mussey, M.D., Professor of Anatomy, Surgery, and Obstetrics.

2. Daniel Oliver, M.D., Professor of Physiology, Theory and Practice of Physic, Materia Medica, and Intellectual Philosophy.

3. Rev. Benjamin Hale, M.A., Professor of Chemistry, and Lecturer on Geology and Mineralogy.

Two of the professors teach in the academical as well as in the medical department; the medical course occupying but fourteen weeks, whilst the academical embraces the year, with the exception of the vacations.

The number of medical students at this college, during the session of 1834-5, was 106; number of graduates, twenty-eight.

### V. UNIVERSITY OF MARYLAND. (BALTIMORE.)

This school is considered to owe its origin mainly to Dr. John B. Davidge, who, in the year 1804, commenced a course of lectures, in Baltimore, on midwifery, to a class of six students. The year following, he lectured also on anatomy and surgery, to a class of seven students; and, in 1806, to a class of nine. In 1807, Dr. Cooke, of Virginia, and Dr. Shaw, of Maryland, united with Dr. Davidge to form a medical school in Baltimore, and lectures were given on the different branches of medicine. The same year, they petitioned the legislature of Maryland for a charter, which was granted, and the school became regularly organized, by the title of the "College of Medicine of Maryland." In the sixteenth section of the charter it was enacted "that, until further arrangements be made by the regents of the said college, John B. Davidge, M.D., and James Cocke, M.D. shall be joint professors of anatomy, surgery, and physiology; George Brown, M.D. professor of the practice and theory of medicine; John Shaw, M.D., professor of chemistry; Thomas E. Bond, M.D., professor of materia medica; and William Donaldson, M.D., professor of the institutes of medicine. Two of these gentlemen, whose solicitude for the interests of science led them to give their influence for the creation of a medical school in Maryland, had no desire to engage in the duties of teaching, and the ill health of another required him to retire to a country residence; so that, in the year 1809, the professors were Davidge, Cooke, Potter, De Butts, and Baker. In 1810, the legislature enlarged the college to a university, by authorizing the formation of three other colleges, and ordered that the four colleges be styled the "University of Maryland."

The faculty at present consists of six professors, in the following order of appointment:

1. Nathaniel Potter, M.D., Professor of Pathology and the Theory and Practice of Physic.

2. Richard Wilmot Hall, M.D., Professor of Midwifery, and the Diseases of Women and Children.

3. Nathan R. Smith, M.D., Professor of the Principles and Practice of Surgery.

* Dr. Jackson has very recently resigned, and his place has not yet been supplied.

4. Julius T. Ducatel, M.D., Professor of Chemistry and Pharmacy.
5. Eli Geddings, M.D., Professor of Anatomy and Physiology.
6. Robley Dunglison, M.D., Professor of Therapeutics, Materia Medica, Hygiène, and Medical Jurisprudence.*

H. Willis Baxley, M.D., Dissector and Demonstrator of Anatomy.

The number of matriculates, session 1835-6, was 120; and of graduates, 46.

### VI. COLLEGE OF PHYSICIANS AND SURGEONS OF THE WESTERN DISTRICT OF THE STATE OF NEW YORK. (FAIRFIELD.)

In the year 1812, this college was instituted by the regents of the University of the State of New York, and placed under the direction of a board of trustees. In the year following, the school was organized by the trustees, with five professorships. The present professors are:

1. W. Willoughby, M.D., President, and Emeritus Professor of Obstetrics.
2. James Hadley, M.D., Professor of Chemistry.
3. James M'Naughton, M.D., Professor of Anatomy and Physiology.
4. Theodoric Romeyn Beck, M.D., Professor of Materia Medica and Medical Jurisprudence.
5. John Delamater, M.D., Professor of the Theory and Practice of Physic.
6. Rueben D. Mussey, M.D., Professor of Surgery and Obstetrics.

The number of students, session 1834-5, was 217.

The college is in Herkimer county, seventy-six miles W.N.W. from Albany.

### VII. YALE COLLEGE, CONNECTICUT. (NEW HAVEN.)

The medical school of Yale College was incorporated by the legislature in the year 1810, and established at New Haven, Connecticut. The lectures did not commence until 1813.

The following gentlemen constitute the present faculty:

1. Thomas Hubbard, M.D., Professor of the Principles and Practice of Surgery.
2. Eli Ives, M.D., Professor of the Theory and Practice of Medicine.
3. B. Silliman, M.D., Professor of Chemistry and Pharmacy.
4. William Tully, M.D., Professor of Materia Medica and Therapeutics.
5. J. Knight, M.D., Professor of Anatomy and Physiology.
6. Timothy P. Beers, M.D., Professor of Obstetrics.

The number of students, session 1834-5, was 64; number of graduates, 17.

### VIII. TRANSYLVANIA UNIVERSITY. (LEXINGTON.)

The medical department of this university was instituted at Lexington, Kentucky, in 1817, and commenced its operations in November of that year. Its rise has been most rapid, and it is now next to the Philadelphia school in point of numbers. The following is the list of students and graduates since its commencement:

| Session. | Students. | Graduates. | Session. | Students. | Graduates. |
|---|---|---|---|---|---|
| 1819-20 | 37 | 7 | 1829-30 | 199 | 81 |
| 1820-21 | 93 | 13 | 1830-31 | 210 | 52 |
| 1821-22 | 138 | 37 | 1831-32 | 215 | 74 |
| 1822-23 | 171 | 51 | 1832-33 | 222 | 69 |
| 1823-24 | 200 | 47 | 1833-34 | 262 | 66 |
| 1824-25 | 234 | 57 | 1834-35 | 259 | 83 |
| 1825-26 | 281 | 65 | 1835-36 | 261 | 75 |
| 1826-27 | 190 | 53 | | | |
| 1827-28 | 152 | 53 | | 3,330 | 923 |
| 1828-29 | 206 | 40 | | | |

* This chair has been recently vacated by the appointment of Professor Dunglison to the chair of "Institutes of Medicine and Medical Jurisprudence," in the Jefferson Medical College.

*Present Faculty:*

1. Benjamin Winslow Dudley, M.D., Professor of Anatomy and Surgery.
2. Charles Caldwell, M.D., Professor of the Institutes and Clinical Practice, and of Medical Jurisprudence.
3. John Esten Cooke, M.D., Professor of the Theory and Practice of Medicine.
4. William Hall Richardson, M.D., Professor of Obstetrics and the Diseases of Women and Children.
5. Charles Wilkins Short, M.D., Professor of Materia Medica and Medical Botany.
6. Lansford Pitts Yardell, M.D., Professor of Chemistry and Pharmacy.

Robert Peter, M.D., assistant Professor of Chemistry.

### IX. MEDICAL COLLEGE OF OHIO. (CINCINNATI.)

This college was established at Cincinnati in 1818; but it has experienced many changes, and the lectures were suspended for a session. A new charter was, however, obtained from the legislature; since which time the number attending the school has, with the exception of one or two years, progressively increased. During the last year a new school was established in Cincinnata, through the agency of some who were previously prominent professors in the Medical College of Ohio. The present professors are:

| | |
|---|---|
| 1. Jedediah Cobb, M.D. . . | Anatomy and Physiology. |
| 2. John Locke, M.D. . . . | Chemistry and Pharmacy. |
| 3. Alban G. Smith, M.D. . . | Surgery. |
| 4. James C. Cross, M.D. . . | Materia Medica. |
| 5. John Moorhead, M.D. . . | Obstetrics, and the Diseases of Women and Children. |
| 6. John Eberle, M.D. . . . | Theory and Practice of Medicine. |
| 7. John T. Shotwell, A.B. M.D. | Adjunct Professor of Anatomy. |

Number of students during the session of 1835-6, about 125.

### X. VERMONT ACADEMY OF MEDICINE. (CASTLETON.)

This institution was established at Castleton, Vermont, under the charter of Middleburg College, in 1818.

The number of students, session 1834-5, was sixty-two.

### XI. MEDICAL SCHOOL OF MAINE. (BRUNSWICK.)

This school was established, in the year 1820, at Brunswick, under the charter of Bowdoin College. The following is the list of professors:

1. Jedediah Cobb, M.D., Professor of Anatomy and Surgery.
2. William Perry, M.D., Professor of the Theory and Practice of Physic.
3. James M'Keen, M.D., Professor of Obstetrics and Medical Jurisprudence.
4. Parker Cleaveland, M.D., Professor of Chemistry and Materia Medica.

The number of students, during the session of 1834-5, was eighty-six.

### XII. BERKSHIRE MEDICAL INSTITUTION. (PITTSFIELD.)

This institution was established at Pittsfield, Massachusetts, in 1822, under the charter of Williams College, situated at Williamstown, in that state.

The following professors constitute the faculty:

1. H. H. Childs, M.D., Professor of the Theory and Practice of Medicine and Obstetrics.
2. E. Bartlett, M.D., Professor of Pathological Anatomy and Materia Medica.
3. C. Dewey, M.D., Professor of Botany, Chemistry, and Natural Philosophy.
4. W. Parker, M.D., Professor of Anatomy, Surgery, and Physiology. (?)
5. John Frissell, A.M., Demonstrator of Anatomy.

The number of students in attendance during the session of 1835-6, was one hundred. Number of graduates, session 1834-5, twenty-seven.

XIII. MEDICAL COLLEGE OF SOUTH CAROLINA. (CHARLESTON.)

In the year 1824, the Medical College of South Carolina was established at Charleston. This school proceeded in prosperity until within the last few years, when dissensions arose between the trustees and the faculty: the latter resigned their chairs, and having obtained, in 1832, a charter from the state, commenced a new school, the "Medical College of the State of South Carolina." Since the secession of the old professors, several changes have been made. The following professors constitute the present faculty of the college:

1. William Hume, M.D., Professor of Anatomy.
2. E. Harry Deas, M.D., Professor of Surgery.
3. Thos. Y. Simons, M.D., Professor of the Theory and Practice of Medicine.
4. Francis Y. Porcher, M.D., Professor of Obstetrics and Diseases of Women and Children.
5. Henry Alexander, M.D , Professor of Materia Medica.
6. Charles Davis, M.D., Professor of Chemistry.

The number of students in attendance during the session of 1834-5, is stated to have been eighteen.

XIV. JEFFERSON COLLEGE, PHILADELPHIA. (CANONSBURG.)

The medical school of Jefferson College, which is seated at Canonsburg, in the western part of Pennsylvania, was established at Philadelphia, in the year 1824. Within the last four years the rise of this institution has been unexampled. In the session of 1832-3, there were only ninety-six students; in 1833-4, 172; in 1834-5, 233; and, in 1835-6, 364. In the first of these years, the number of graduates was nineteen; in the last, 134.

The following is the list of professors:

1. Granville Sharp Pattison, M.D., Professor of Anatomy.
2. George M'Clellan, M.D., Professor of Surgery.
3. John Revere, M.D., Professor of the Principles and Practice of Physic.
4. Samuel Colhoun, M.D., Professor of Materia Medica and Pharmacy.
5. Jacob Green, M.D., Professor of Chemistry.
6. Samuel M'Clellan, M.D., Professor of Midwifery and the Diseases of Women and Children.*

XV. UNIVERSITY OF VIRGINIA. (CHARLOTTESVILLE.)

When this school was first established, but one medical professor was appointed, to whom every branch of medicine was assigned, except chemistry. The author of this sketch was sole professor from 1825, the year in which the lectures were first delivered, until the session of 1827-8; when, on his proposition, the visiters assigned the departments of practical anatomy and surgery to a demonstrator, and that of materia medica (with his approbation) to the professor of chemistry. Unlike the mass of medical schools, the session of the medical department of the University of Virginia is of the same length as the academic session, ten months. The following gentlemen compose the faculty:

1. John P. Emmet, M.D., Professor of Chemistry and Materia Medica.
2. Alfred T. Magill, M.D., Professor of the Theory and Practice of Medicine, Obstetrics, and Medical Jurisprudence.
3. Augustus L. Warner, M.D., Professor of Anatomy, Physiology, and Surgery.

The number of students in attendance during the session of 1835-6, was sixty-three.

XVI. WASHINGTON MEDICAL COLLEGE. (BALTIMORE.)

This institution, which is an offset of the Washington College, Washington, Pennsylvania, after a career of six years' duration, obtained, in 1833-4, an act

* A new chair has recently been added to this school, "Institutes of Medicine and Medical Jurisprudence," to which Professor Dunglison has been appointed.

of incorporation from the state of Maryland, empowering the authorities of the college to confer degrees in medicine.

The present faculty are:

1. James H. Miller, M.D., Professor of Anatomy, Physiology, and Pathology.
2. Samuel H. Jennings, M.D., Professor of Materia Medica, Therapeutics, Hygiène, and Medical Jurisprudence.
3. William H. Handy, M.D., Professor of Obstetrics and the Diseases of Women and Children.
4. John C. S. Monkur, M.D., Professor of the Theory and Practice of Medicine.
5. John P. Mettauer, M.D., Professor of Surgery and Surgical Anatomy.
6. Edward Foreman, M.D., Lecturer on Chemistry.

Washington R. Handy, M.D., Demonstrator of Anatomy.

The number of students in attendance during the last session was about twenty.

### XVII. MEDICAL COLLEGE OF GEORGIA. (AUGUSTA.)

In the year 1828, a medical institution was commenced at Augusta, Georgia, under the name of the "Medical Academy," by Dr. Antony. As this did not succeed, or at least was not encouraged by a reciprocity of favours from the other medical establishments of the country, the charter was extended by the legislature of Georgia, in the session of 1829-30, so as to enable the college to grant degrees, under the same regulations as other medical colleges. The first course of lectures was delivered in the winter of 1832-3.

*Present Faculty:*

1. Alexander Cunningham, M.D., Professor of the Theory and Practice of Medicine.
2. Paul F. Eve, M.D., Professor of Surgery.
3. A. Dugas, M.D., Professor of Anatomy.
4. Joseph Eve, M.D., Professor of Materia Medica.
5. Milton Antony, M.D., Professor of Obstetrics, &c.
6. Lewis Ford, M.D., Professor of Chemistry.

### XVIII. MEDICAL COLLEGE OF THE STATE OF SOUTH CAROLINA. (CHARLESTON.)

In consequence of difficulties originating between the Faculty of the Medical College of South Carolina, (No. XIII.) and the Medical Society of the State, the governing body, in the year 1832, the former resigned their professorships, and obtained from the legislature a charter for a college under the above title. This was organized in 1833.

*Present Professors.*

1. G. Edwards Holbrook, M.D., Professor of Anatomy.
2. John Wagner, M.D., Professor of Surgery.
3. S. Henry Dickson, M.D., Professor of the Institutes and Practice of Medicine.
4. Henry R. Frost, M.D., Professor of Materia Medica.
5. G. A. Shepard, M.D., Professor of Chemistry.
6. Thomas G. Prioleau, M.D., Professor of Obstetrics, &c.
7. James Moultrie, M.D., Professor of Physiology.
8. John Bellinger, M.D., Demonstrator of Anatomy.

The number of students in attendance in 1835-6, was 129; number of graduates, session 1834-5, 38.

### XIX. MEDICAL COLLEGE OF LOUISIANA. (NEW ORLEANS.)

This school was instituted in the autumn of 1834, in New Orleans, with the above title, and it has since been endowed by the state legislature with corporate privileges.

The following professors compose the faculty:

1. Charles A. Lazenberg, M.D., Professor of the Principles and Practice of Surgery.
2. Edward H. Barton, M.D., Professor of the Theory and Practice of Medicine and Clinical Practice.
3. W. Byrd Powell, M.D., Professor of Chemistry and Pharmacy.
4. J. Harrison, M.D., Professor of Physiology and Pathology.
5. J. Monroe Mackie, M.D., Professor of Materia Medica, Therapeutics, and Medical Jurisprudence.
6. Thomas R. Ingalls, M.D., Professor of Obstetrics, and Diseases of Women and Children.
7. C. A. Luzenberg, M.D., Professor of Anatomy, (*ad interim*).

XX. MEDICAL INSTITUTION OF GENEVA COLLEGE, NEW YORK. (NEW YORK.)

The trustees of Geneva College, at Geneva, on the Seneca Lake, have established a Medical department; the number of students at which, during the last session, was sixty-eight; and of graduates, six.

*Present Professors.*

1. E. Cutbush, M.D., Professor of Chemistry.
2. Thomas Spencer, M.D., Professor of the Institutes and Practice of Medicine.
3. W. Parker, M.D., Professor of Anatomy and Physiology.
4. John George Morgan, M.D., Professor of the Principles and Practice of Surgery.
5. Charles B. Coventry, M.D., Professor of Obstetrics and Materia Medica.
6. A. Coleman, M.D., Professor of Medical Jurisprudence and Botany.

XXI. MEDICAL DEPARTMENT OF CINCINNATI COLLEGE. (CINCINNATI.)

The medical department of this college was instituted last year, under the following professors.

1. Joseph A. M'Dowell, M.D., Professor of Special and Surgical Anatomy.
2. Samuel D. Gross, M.D., Professor of General and Pathological Anatomy, Physiology, and Medical Jurisprudence.
3. William Parker, M.D., Professor of Surgery.
4. Landon C. Reeves, M.D., Professor of Obstetrics, and the Diseases peculiar to Women and Children.
5. James B. Rogers, M.D., Professor of Chemistry and Pharmacy.
6. John P. Harrison, M.D., Professor of Materia Medica.
7. Daniel Drake, M.D., Professor of the Theory and Practice of Medicine.

John L. Reddel, M.A., Adjunct Professor of Chemistry and Lecturer on Botany.

The number of students in attendance during the course was sixty-six.

XXII. SCHOOL OF MEDICINE AT WOODSTOCK, VERMONT. (WOODSTOCK.)

This school was incorporated by the legislature of Vermont, in October, 1835, with the power of conferring degrees. It is connected, like the Vermont Academy of Medicine (No. X.) with Middleburg College.

*Present Faculty.*

1. H. H. Childs, M.D., Professor of the Theory and Practice of Medicine, and Obstetrics.
2. William Parker, M.D., Professor of Physiology and Surgery (?)
3. David Palmer, M.D., Professor of Chemistry and Materia Medica.
4. Robert Watts, jun., M.D., Professor of Anatomy.
5. Norman Williams, A.M., Professor of Medical Jurisprudence.

Otes Perham, Demonstrator of Anatomy.

XXIII. WILLOUGHBY UNIVERSITY, LAKE ERIE. (WILLOUGHBY.)

The medical department of this university was founded a short time ago. We know not the names of the professors; but, according to the Western Journal of the Medical and Physical Sciences, (for April, 1836,) it has five. The catalogue for 1835-6, presents the names of twenty-three students; and of five graduates.

There are, consequently, in the United States, not fewer than twenty-three colleges capable of conferring medical degrees, and these are attended during the session by not fewer than 2,500 students; of whom, at least five or six hundred may receive degrees. This may seem an inordinate supply, but it must be borne in mind that the division of the profession is not the same in the United States as in England. The apothecary of the United States corresponds to the *Pharmacien* of France; he neither visits the sick, nor prescribes at the counter. Every Candidate, too, for graduation, is compelled to exhibit his qualifications for practising both medicine and surgery; for, although some may devote themselves more especially to the latter branch, their medical education does not differ from that of the practitioner who confines himself to medicine. They are all educated, in other words, for the general exercise of the duties of their profession.

All the Institutions are organized upon the same general plan, although they may differ in the number of professorships, and in the facilities which they afford for the study of the more practical parts of the profession. In the largest schools of the country, it is expected that the candidate for graduation shall have studied medicine three full years, and it is required that he shall have attended two courses of public lectures on all the branches of the profession at a regularly organized Medical Institution. He is then subjected to a private examination before the Medical Faculty; and, in most of the schools, has to defend, in their presence, an acceptable dissertation on some medical topic. In most of the colleges, too, the candidate must be twenty-one years of age, and of good moral character; and in some he is required to possess an acquaintance with natural philosophy and a knowledge of the principles and construction of the Latin language. In all cases the examinations are in English; and in one school,—the University of Virginia,—the diploma is in that language, and of the simplest construction.

Attached to most of the schools, in the cities more especially, are valuable hospitals, or dispensaries, in which the student has full opportunity for investigating the nature of disease, and the approved methods of management, and for witnessing surgical operations. A year's attendance upon the clinics of an institution of this kind is properly required by most of the schools; and by some the same length of attendance on practical anatomy, in addition to the two courses delivered by the professor of anatomy, is held to be indispensable.

In the University of Virginia, the session of which, as was before remarked, is of ten months' duration, time has been altogether discarded in the estimate of the student's qualifications for a degree, and he is permitted to present himself for examination at the prescribed period of the session, during the first year of his collegiate attendance, if he thinks proper.

The fees for attendance on lectures vary greatly in the different schools. In the oldest institution in the country, the University of Pennsylvania, they amount to 120 dollars (27*l.*) for the course, whilst there are schools in which they do not amount to more than fifty-five dollars, (12*l.*) The fee for the Diploma likewise varies, from forty dollars (University of Pennsylvania,) to five dollars (University of Virginia). In the latter institution, it was thought that the student should be entitled to his certificate of proficiency, or diploma, when he afforded the faculty satisfactory evidences thereof; and, accordingly, a small sum was affixed to the document, with the view merely of covering the expenses of parchment, &c.

The mode of teaching is precisely that adopted in the medical institutions of Europe, by lectures, aided (where the subject admits of it,) by demonstrations; for which extensive museums, cabinets of apparatus, &c. afford ample facilities.

The session of the purely Medical Schools generally commences about the first of November, and terminates on the last day of February. The anatomical rooms are sometimes opened earlier. This is the case with the Jefferson College, of Philadelphia, at which lectures are also delivered during the month of October.

*Table of the Dates at which the Lectures commence in the different Institutions for Medical Instruction in the United States.*

| | | |
|---|---|---|
| 1. University of Pennsylvania . . | at Philadelphia, | First Monday in November. |
| 2. College of Physicians and Surgeons, New York . . . . | New York, . | Ditto ditto. |
| 3. Medical School of Harvard . . | Boston, . . | First Wednesday in Novemb. |
| 4. Dartmouth Coll., New Hampshire | Hanover, . . | Last Week in August. |
| 5. University of Maryland . . . | Baltimore, . | Last Monday in October. |
| 6. College of Physicians and Surgeons of the Western District of the State of New York . | Fairfield, . | First Tuesday in October. |
| 7. Yale College, Connecticut . . . | New Haven, | Last week in October. |
| 8. Medical College of Ohio . . . | Cincinnati, . | Last Monday in October. |
| 9. Vermont Academy of Medicine . | Castleton, . | Second Thursday in August, and a spring term beginning on the second Thursday in March. Each session of fourteen weeks. |
| 10. Transylvania University . . . | Lexington, . | First Monday in November. |
| 11. Medical School of Maine . . . | Brunswick, . | Latter end of February. |
| 12. Berkshire Medical Institution, Massachusetts . . . . | Pittsfield, . . | Last Thursday in August. |
| 13. Medical College of South Carolina | Charleston, . | Second Monday in November. |
| 14. Jefferson College . . . . . | Philadelphia, | The regular series, in the first week of November. |
| 15. University of Virginia . . . . | Charlottesville | First of September. |
| 16. Washington Medical College . . | Baltimore, . | Last Monday in October. |
| 17. Medical College of Georgia . . | Augusta, . . | In October. |
| 18. Medical College of the State of South Carolina . . . . | Charleston, . | Second Monday in November. |
| 19. Medical College of Louisiana . . | New Orleans, | First Monday in December. |
| 20. Medical Institution of Geneva College, New York . . | Geneva, . . | First Tuesday in October. |
| 21. Cincinnati College . . . . . | Cincinnati, . | Last Monday in October. |
| 22. School of Medicine at Woodstock, Vermont . . . . | Woodstock, . | Second Thursday in March. |
| 23. Willoughby University . . . . | Willoughby, Lake Erie, . | Beginning of November. |

*Baltimore; June 10th,* 1836. R. D.

